# A Case of Pallister–Killian Syndrome in a Newborn

**DOI:** 10.1155/crig/8819010

**Published:** 2026-05-30

**Authors:** Giulia Di Donato, Chiara Cauzzo, Paola Cicioni, Teresa Topazio, Valentina Chiavaroli, Chiara Palka Bayard De Volo, Elisena Morizio, Francesco Chiarelli, Susanna Di Valerio

**Affiliations:** ^1^ Department of Pediatrics, Gabriele d’Annunzio University of Chieti and Pescara, Chieti, Italy; ^2^ Neonatal Intensive Care Unit, Pescara Public Hospital, Pescara, Italy; ^3^ Genetic Unit, Department of Oral Sciences, Nano and Biotechnologies, Gabriele d’Annunzio University of Chieti and Pescara, Chieti, Italy

**Keywords:** isochromosome 12p, mosaicism, Pallister–Killian syndrome, tetrasomy

## Abstract

**Background:**

Pallister–Killian syndrome (PKS) is a rare disorder caused by tissue‐limited mosaicism tetrasomy of chromosome 12p. Affected newborns show a typical dysmorphic pattern: macrosomia, coarse facies, hypertelorism, small nose with long philtrum, V‐shaped upper lip, low set ears, frontotemporal alopecia, and patchy pigmentary skin and hair anomalies. Seizures and developmental delay, cardiac defects, diaphragmatic hernia, and renal/anal malformations may be associated.

**Case:**

Here, we report the case of a newborn with multiple congenital malformations, later diagnosed with PKS.

**Conclusions:**

Phenotypic and cytogenetic variability of PKS, together with the lack of correlation between tetrasomic cells’ proportion and disease severity, may be challenging for diagnosis. Therefore, a detailed physical examination is mandatory for early clinical suspicion and to guide further investigations. The usefulness of array‐CGH performed on peripheral blood should also be emphasized as a sensitive diagnostic tool.

## 1. Introduction

Pallister–Killian syndrome (PKS) is a rare multisystem developmental disorder, with a peculiar phenotype caused by tissue‐limited mosaicism tetrasomy of the short arm of chromosome 12 (isochromosome 12p) [1]. PKS was described by Pallister in 1977 [2] and later by Killian and Teschler‐Nicola in 1981 [3]. The dysmorphic pattern is characterized by coarse facies, hypertelorism, small nose with long philtrum, V‐shaped upper lip (“Pallister lip”), low‐set ears, broad and short hands and fingers, frontotemporal alopecia, and pigmentary skin anomalies [4]. Areas of hyper‐ or hypopigmentation of skin and hair are often found, reflecting the mosaic distribution of genetic anomalies and hyperdiploid cells [5]. Neonates may experience hypotonia and feeding difficulties. Macrosomia is often present at birth, with late deceleration of postnatal growth. The spectrum of associated manifestations is heterogeneous, including musculoskeletal, ophthalmologic, audiologic, diaphragmatic, cardiac, gastrointestinal, genitourinary anomalies, and anorectal malformations [6]. Some patients can develop severe neurodevelopmental delay, with intellectual disability and seizures, but a mild course of disease has also been described [7].

Here, we report the case of a female newborn with dysmorphic features and multiple congenital malformations, later diagnosed with PKS.

## 2. Case History/Examination

A female infant was born in July 2020 from vaginal delivery at 41 weeks’ gestation at the maternity unit of Pescara Public Hospital, Pescara, Italy. The girl was the first offspring of unrelated healthy parents. The mother was 43 years old. Pregnancy was characterized by maternal hypothyroidism, treated with levothyroxine. Prenatal ultrasound was normal. Family history was negative for congenital malformations.

At delivery, the Apgar score was 8 at 1 min and 9 at 5 min of life. Birth anthropometric parameters were the following: weight 4030 g (95th percentile), length 54 cm (100th percentile), and head circumference 34.5 cm (59th percentile). On neonatal examination, hypertelorism, ogival palate, a white hair wisp, ulnar fingers deviation, and anteriorly displaced anus with perineal fistula were observed.

After birth, the infant showed severe respiratory distress, with development of persistent pulmonary hypertension, requiring intubation and ventilatory support with conventional and nonconventional systems. Antibiotics therapy was started for neonatal sepsis. Gradual improvement in respiratory function and resolution of sepsis allowed for discontinuation of ventilatory support.

Brain ultrasound and magnetic resonance imaging (MRI) showed no alterations. Fundus oculi examination and acoustic otoemissions were normal. Abdominal ultrasound and MRI documented the presence of a cystic lymphangioma and multiple bilateral ovarian cysts. Unilateral hydronephrosis was also diagnosed. Spinal ultrasound examination was performed to exclude the presence of spinal cord anomalies.

**FIGURE 1:**
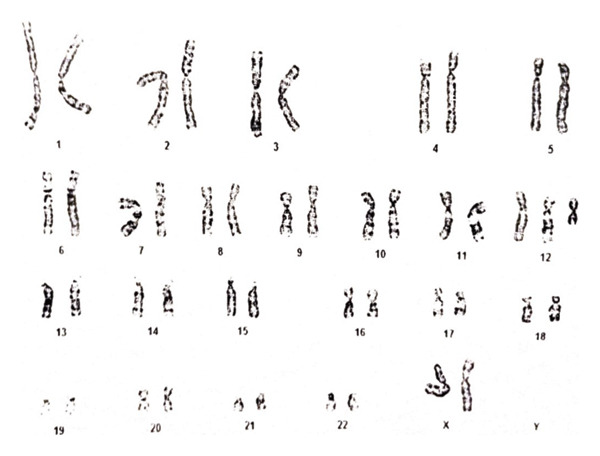
Patient’s karyotype on peripheral blood showing the presence of isochromosome 12p in a single metaphase: mos47,XX,i(12)(p10)[1]/46,XX[99].

**FIGURE 2:**
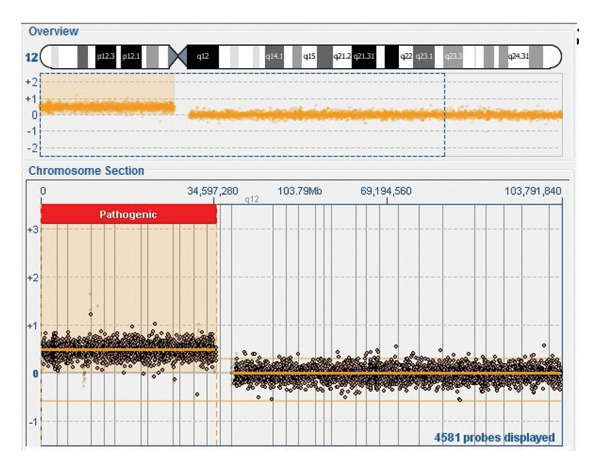
Patient’s CGH‐array on peripheral blood confirmed the presence of 12p13.33p11.1 duplication.

Genetic karyotype on peripheral blood revealed the presence of supernumerary isochromosome 12p, allowing the diagnosis of PKS. This alteration was found in one metaphase out of 100 examined: mos47,XX,i(12)(p10)[1]/46,XX[99] (Figure 1). Array comparative genomic hybridization (array‐CGH) was performed using a v2 4 × 180 K CytoSure Oligo ISCA OGT with a resolution of ∼100 KB (Oxford Gene Technology), according to the recommendations of the manufacturer. It confirmed the presence of a gain on chromosome 12 ranging from 12p13.33 to 12p11.1 at position 0–34,597,280 (Figure 2). The slides were scanned with an InnoScan 710 Microarray Scanner, and captured images were analyzed with CytoSure Interpret software Version 4.10. Usually, probes are distributed around the +2 line in cases of trisomy, around the +3 line in cases of tetrasomy, and around Line 0 under normal conditions. A mosaic state is considered when the probes are restricted between these two lines (0–2). When probes are near Line 0, the explanation is difficult. Log ratio calculates the relationship between patient DNA signals and control DNA signals (*X* = [log2 (number of patient copy/number of control copy)]. In normal state, it is balanced and is 0: [log2 (2/2)] = 0. In the case of a duplication of 100% of cells, the value will be +0.58 [log2 (3/2)] = 0.58). When there is a mosaic state, the obtained values are different. Assuming a linear mixture of diploid (CN = 2) and tetrasomic (CN = 4) cells, the expected log2 ratio is given by LR = log2 (1+*f*), where *f* is the fraction of tetrasomic cells. Accordingly, *f* = 2^LR^−1. Visually, in the present case, the probes band is around LR +0.30/0.35. In case of a tetrasomy, the number of patient copies is 4, and so if LR is 0.30, f will be 0.23, and if LR is 0.35, f will be 0.27. In conclusion, the percentage of i(12p) is approximately 25%–30%.

At 30 days of life, the girl was discharged in good general clinical condition, with no clinically evident neurological abnormalities, and was scheduled for neurological follow‐up. Since the first follow‐up visits at 3 months of age, the infant showed signs of hypotonia and reduced motility. At the 6‐month follow‐up visit, she presented with marked hypotonia, severe neurological impairment, and blindness. Acoustic evoked potentials were normal. No seizures developed.

## 3. Discussion

PKS is a rare, sporadic genetic disorder. A prevalence of 1/20.000 (higher in females) has been reported, but it seems to be underestimated, due to the difficulty of cytogenetic diagnosis from peripheral blood [8]. Isochromosome 12p is a chromosome composed of mirror images of only the p arm of the chromosome. It may be caused by a transverse division of the centromere during the meiosis/mitosis. Alternatively, it may result from a U‐type strand exchange: Adouble‐strand break in a chromosome is repaired by fusing the two sister chromatids (or homologous chromosomes) in a “U” shape, leading to an inverted duplication and a terminal deletion. It may occur in the premeiotic gamete during meiotic cell division or in the postzygotic cell division stage leading to mosaicism. Isochromosomes are often supernumerary, presumably because the loss of even one copy of a chromosome arm can be lethal. The isochromosome 12p seen in PKS is always present in a mosaic form, demonstrating the example of an abnormality that is lethal when constitutional but tolerated if the abnormal chromosome can be eliminated from key tissues [9]. The mechanism by which isochromosomes are formed and the stage at which this occurs are greatly debated and still not understood. Molecular analysis of isochromosomes shows that they predominantly originate through maternal meiosis II nondisjunction, followed by a rearrangement or centromeric misdivision [10]. Isochromosome 12p can be detected prenatally in chorionic villus and amniotic fluid cell samples or later in lymphocytes and skin fibroblast cultures [11, 12]. PKS genetic diagnosis is challenging, due to the tissue‐specific mosaicism of this condition: 12p tetrasomy detection rate is 0%–2% in lymphocytes, 50%–100% in fibroblasts and chorionic villi, and 100% in amniocytes and bone marrow [13]. Conventional cytogenetic methods might not reveal the presence of mosaicism for isochromosome 12p on peripheral blood, since cultured lymphocytes may undergo a negative selection for chromosome abnormalities. Isochromosome 12p has also been reported to be unstable with age in blood but constant in skin fibroblasts [14]. Furthermore, higher percentage of normal cells and lower degree of mosaicism are present in lymphocytes as compared to fibroblasts. Therefore, cytogenetic analysis (G‐banded karyotype) and fluorescence in situ hybridization (FISH) of skin fibroblasts are the preferred diagnostic tests [15]. However, skin biopsy is an invasive method, and the detection rate may be reduced in the nonpigmented regions. Noninvasive techniques, such as buccal swabs, are a valid and promising tool, with a 50%–100% detection rate [16]. Array‐CGH and single‐nucleotide polymorphism array (SNP array) have improved the detection of tetrasomy 12p in peripheral blood. Array‐CGH on blood sample has a high sensitivity in the diagnosis of mosaicism of isochromosome 12p [17, 18]. It presents several advantages, since it provides results on extracted genomic DNA and does not require cell cultures, better reflecting the value of mosaicism. Moreover, many cells are analyzed simultaneously, and different cell types are analyzed. Also, it can simultaneously analyze cells in different cell cycles. [19]. Therefore, it is superior to standard cytogenetic techniques in detecting mosaic i(12p) [7]. In our patient, the typical genetic alteration was found only in one metaphase out of 100 examined at conventional karyotype on blood. This does not meet the standard criteria for diagnosis of mosaicism. Indeed, an evaluation of 30 cells is recommended to exclude the presence of mosaicism at a level of 10% or greater, with 95% confidence [20].

Despite this, the clinical features and the presence of the isochromosome 12p in a single cell were highly suggestive of PKS. Consequently, further testing was necessary to confirm this suspect.

Array‐CGH on peripheral blood confirmed the presence of 12p13.33p11.1 duplication and revealed that 25%–30% of cells were positive for trisomy 12p. So, at the end, this single cell was diagnostic despite not meeting criteria. This case highlights the importance of identifying key clinical signs that guide clinicians toward the most appropriate diagnostic tests.

Prenatal diagnosis of PKS is often incidental at karyotyping of amniotic fluid cells in case of advanced maternal age, increased nuchal translucency thickness or fetal anomalies [21]. No specific ultrasound fetal anomalies have been associated with PKS. However, decreased fetal movements, macrosomia, polyhydramnios, diaphragmatic hernia, rhizomelic limb shortening, and associated ductus venosus agenesis are the most reported prenatal ultrasound abnormalities [22]. Fetal growth pattern is typical, with increased biparietal diameter and head circumference (> 90th percentile) and delayed femoral growth (< 10th percentile) [23]. Array‐CGH on DNA extraction from uncultured amniocytes may allow the final diagnosis. In our patient, prenatal tests on amniotic fluid were not performed, and prenatal ultrasound examination was normal.

PKS is defined by the association of a dysmorphic face with pigmentary skin/hair anomalies, hypotonia, neurodevelopmental delay, and seizures [23]. Namely, typical features include frontal bossing, thin eyebrows, hypertelorism, small nose with flat philtrum, low‐set dysplastic ears, V‐shaped upper lip, and micrognathia. The dysmorphic pattern is age‐dependent, with progressive coarsening of the face and development of macroglossia and prominent chin and lips [6, 7]. Oral cavity anomalies, like alveolar ridge or gingival hypertrophy and altered teeth eruption, may be associated [24]. Pigmentation anomalies of skin and hair are often patchy and indicative of the mosaic chromosomal abnormality: They are not always evident, so that Wood lamp examination is indicated in case of suspicion. Some patients have rhizomelic or proportionate limb shortening; hands, fingers, feet, and toes are proportionately small, with associated lymphedema and increased soft tissue of the extremities. Supernumerary nipples, cryptorchidism, and hypospadias are often described. Marked hypotonia can be associated with feeding difficulties. Polydactyly, joint contractures, and hip dislocations are frequently reported in PKS. Radiological examinations may help the diagnosis, due to the typical presence of metaphyseal enlargement of long bones, ovoid vertebral bodies, and delayed axial and pubic bone endochondral ossification [25].

Growth shows a characteristic pattern, with macrosomia at birth and postnatal decline in growth velocity. Anterior fontanel closure is delayed. Pubertal timing is also delayed in boys [26]. Our patient presented typical dysmorphic features, such as hypertelorism, ogival palate, pigmentary hair anomalies, and ulnar fingers deviation. Macrosomia was also diagnosed at birth.

Congenital heart defects, diaphragmatic hernia, and anal defects are frequently associated with PKS, while gastrointestinal and genitourinary malformations are rarely reported. Our patients showed typical associated anomalies, like anteriorly displaced anus and perineal fistula. She also presented abdominal cystic lymphangioma and multiple bilateral ovarian cysts, rarely reported in literature.

Sensorineural, conductive, or mixed hearing loss occurs in 77% of PKS patients. Ocular involvement affects 87% of patients with strabismus, nystagmus, myopia, or complete blindness, like in our patient [8]. Seizures and structural brain malformations are often seen in PKS. Patients can present with myoclonic, tonic, tonic–clonic seizures, or absences, mainly in the first 4 years of life. Nonepileptic paroxysmal events are often associated, frequently requiring combined therapy [27]. PKS also includes autonomic dysfunction, characterized by hypo/anhidrosis and hyperventilation. Many patients develop profound intellectual disability, while others have a mild or moderate developmental delay, with typical self‐stimulatory or self‐injurious behaviors [7]. Common brain MRI alterations include brain atrophy, polymicrogyria, cortical malformations, white matter disease, corpus callosum dysgenesis, brachium pontis signal abnormalities, spot calcifications of the perisylvian region, and vermian dysplasia [28, 29]. Brain MRI was normal in our patient, and she was not affected by seizures and autonomic symptoms. However, she developed severe developmental delay.

PKS phenotype is similar to trisomy 12p, resulting from balanced parental translocations. Patients with trisomy 12p lack skin pigmentation anomalies and major malformations and have a better neurologic outcome. This syndrome should be considered in the differential diagnosis [30].

In conclusion, the phenotypic and cytogenetic variability of PKS, together with the lack of correlation between the proportion of tetrasomic cells and disease severity, may pose challenges for diagnosis. This case highlights the importance of performing a detailed physical examination for early clinical suspicion, particularly in neonates with multiple congenital malformations. It also emphasizes the usefulness of array‐CGH performed on peripheral blood as a sensitive and less invasive diagnostic tool, especially when carried out early in life prior to skin biopsy. Neuroimaging and skeletal radiological evaluation may further support the diagnosis in subtle cases. A strict follow‐up is mandatory, because of the associated risk of neurodevelopmental impairment.

## Author Contributions

All authors contributed to the work. Giulia Di Donato and Chiara Cauzzo wrote the manuscript. Susanna Di Valerio, Valentina Chiavaroli, Paola Cicioni, and Teresa Topazio examined the patient. Chiara Palka Bayard De Volo and Elisena Morizio performed the genetic tests. Giulia Di Donato and Valentina Chiavaroli were involved in the literature search and drafting of the paper. Susanna Di Valerio and Francesco Chiarelli coordinated and approved the final version of the manuscript.

## Funding

The authors have nothing to report. Open access publishing facilitated by Universita degli Studi Gabriele d’Annunzio Chieti Pescara, as part of the Wiley ‐ CRUI‐CARE agreement.

## Disclosure

An earlier version of this manuscript has been presented as abstract at the SIMP Agora 2022: 23rd National Congress of the Italian Society of Perinatal Medicine, September 29th‐October 1st, 2022, Genoa, Italy. The content has not been published or submitted for publication elsewhere.

## Ethics Statement

Consent was obtained from the parents regarding the publication of the case and images. This report does not contain any personal information that could lead to the identification of the patient.

## Conflicts of Interest

The authors declare no conflicts of interest.

## Data Availability

The clinical and genetic data used to support the findings of this study are included within the article.
